# Fractalkine/CX_3_CR1 in Dilated Cardiomyopathy: A Potential Future Target for Immunomodulatory Therapy?

**DOI:** 10.3390/cells12192377

**Published:** 2023-09-28

**Authors:** Visvesh Jeyalan, David Austin, Shu Xian Loh, Vincent Kharisma Wangsaputra, Ioakim Spyridopoulos

**Affiliations:** 1 Academic Cardiovascular Unit, The James Cook University Hospital, Middlesbrough TS4 3BW, UK; v.jeyalan@nhs.net (V.J.); david.austin@nhs.net (D.A.); 2 Translational and Clinical Research Institute, Newcastle University, Newcastle upon Tyne NE2 4HH, UK; v1ncekharisma@gmail.com; 3 Population Health Science Institute, Newcastle University, Newcastle upon Tyne NE1 7RU, UK; 4 Department of Cardiology, Freeman Hospital, Newcastle upon Tyne NHS Foundation Trust, Newcastle upon Tyne NE7 7DN, UK; shuxian.loh1@nhs.net; 5 Faculty of Medicine, Universitas Indonesia, Central Jakarta 10430, Indonesia

**Keywords:** dilated cardiomyopathy, CX3CR1, fractalkine, T lymphocytes, cytomegalovirus

## Abstract

Dilated cardiomyopathy (DCM) is a cardiac condition with structural and functional impairment, where either the left ventricle or both ventricular chambers are enlarged, coinciding with reduced systolic pump function (reduced ejection fraction, rEF). The prevalence of DCM is more than 1:250 individuals, and mortality largely due to heart failure in two-third of cases, and sudden cardiac death in one-third of patients. Damage to the myocardium, whether from a genetic or environmental cause such as viruses, triggers inflammation and recruits immune cells to the heart to repair the myocardium. Examination of myocardial biopsy tissue often reveals an inflammatory cell infiltrate, T lymphocyte (T cell) infiltration, or other activated immune cells. Despite medical therapy, adverse outcomes for DCM remain. The evidence base and existing literature suggest that upregulation of CX_3_CR1, migration of immune cells, together with cytomegalovirus (CMV) seropositivity is associated with worse outcomes in patients with dilated cardiomyopathy. We hypothesise that this potentially occurs through cardiac inflammation and fibrosis, resulting in adverse remodelling. Immune modulators to target this pathway may potentially improve outcomes above and beyond current guideline-recommended therapy.

## 1. Introduction

A reduced ejection fraction, or HFrEF, and enlargement of either the left ventricle or both ventricular chambers are hallmarks of dilated cardiomyopathy (DCM), a disorder that affects both the structure and function of the heart. Clinically, patients display heart failure symptoms in the absence of congenital, valvular, or coronary artery disease. DCM is categorised as either inherited (familial) or acquired (non familial) by the European Society of Cardiology (ESC). Male patients experience DCM more frequently (2:1 to 3:1) and with worse outcomes than female patients [1]. More than 1 in 250 people have DCM; progressive heart failure (70%) and sudden cardiac death (30%) account for the majority of deaths [2]. There is still a significant mortality rate even with recent improvements in the guideline-recommended heart failure treatment. Remodelling and fibrosis leading to the development of scar tissue promote the distortion of the ventricle into a spherical shape, causing the chambers to dilate, and as a result, the stroke volume, cardiac output, ventricular filling, and left ventricular end diastolic pressure (LVEDP) are all affected negatively. Early intervention is difficult before permanent damage has taken place since heart failure symptoms frequently appear gradually after the beginning of cardiac damage.

**Figure 1:**
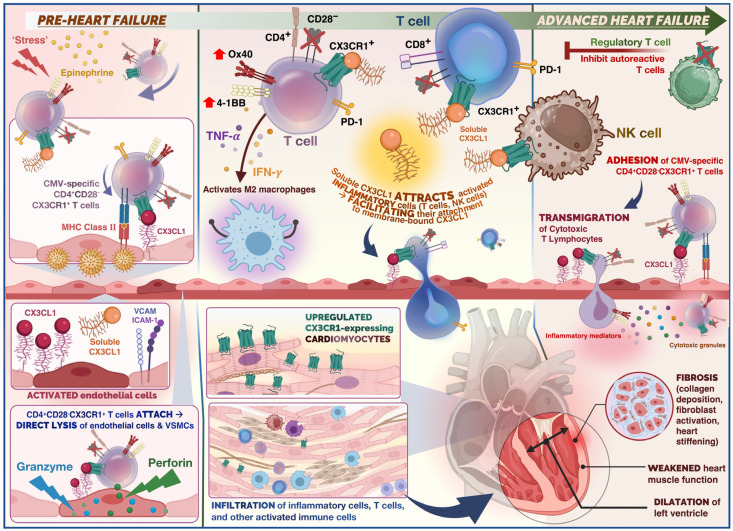
Summary of the role of immune cells in the cardiovascular disease continuum from the pre-heart failure to advanced heart failure stage in dilated cardiomyopathy (DCM). Physiological stress and release of epinephrine in the acute phase (pre-heart failure), triggers release of CX3CR1+ T cells. In the case of stress-induced viral reactivation, rapid mobilisation for CMV-specific T cells occurs. CX3CR1+ CMV-specific CD4+ T cells would adhere to endothelium, and undergo antigen recognition, leading to activation of cytotoxic effector cells. Direct lysis of endothelial cells and vascular smooth muscle cells (VSMCs) occurs (mediated by granzymes and perforin). Release of cytokines such as TNF-α and IFN-γ as well as chemokines from CMV-specific CD4+ T cells potentially attracts further immune cells such as polarised macrophages and NK cells through endothelial induction of fractalkine, which may enhance endothelial injury. The CX3CR1– regulatory T cells (Tregs) are capable of inhibiting autoreactive T cells. Thus, administration of Tregs serves as a potential therapeutic target to promote immune tolerance. Soluble fractalkine binds to its chemokine receptor on cytotoxic T cells and NK cells which facilitates adhesion and transmigration across the endothelium. Infiltrating T cells and inflammatory cells release inflammatory mediators and cytotoxic granules which promote remodelling, collagen deposition, and fibrosis. This ultimately results in weakening of the ventricular heart muscle and ventricular dilatation.

Damage to the myocardium, whether caused by genetic or environmental factors, induces inflammation and recruits immune cells to repair the myocardium; the most common causes of inflammatory DCM are infections and autoimmunity [3]. Examining myocardial biopsy tissue typically reveals an inflammatory cell infiltrate, such as CD3 T lymphocyte infiltration, or other activated immune cells, such as M2 macrophages and B lymphocytes, in autoimmune diseases such as rheumatoid arthritis, systemic sclerosis, systemic lupus erythematosus, and Dresslers syndrome to name a few. All of these are capable of releasing cytokines, including transforming growth factor-1 (TGF1), interleukin-4 (IL-4), IL-1, IL-17A, IL-33, and tumour necrosis factor (TNF), which can promote remodelling, collagen deposition, and fibrosis [4]. Fibrosis results from inflammation at the site of tissue injury and is the distinguishing pathological feature of DCM, in addition to dilatation [5] (see Figure 1).

Similar to healing post-myocardial infarction, fibrotic scar tissue ultimately replaces damaged tissue, stiffening the heart through fibrosis, and eventually exacerbating the progression to dilation and heart failure. The absence of treatment guidelines for inflammatory DCM causes necessitates further clinical investigation. Infections caused by viruses are a common cause of myocarditis, which can contribute to inflammatory DCM in certain individuals [6].

While DCM accounts for half of the cases of non-ischemic cardiomyopathies, this review will focus primarily on patients with idiopathic DCM (30–40%) and auto-immune disease background (5–10%) who do not respond well to standard treatment (see Table 1 for causes) [7]. Both are believed to have a significant inflammatory component that could be targeted by specific immune modulators. A worse prognosis is associated with an increased myocardial immune response in patients with DCM [8]. The association between M2 macrophages and collagen production suggests that ventricular remodelling in DCM may be associated with the phenotypic polarisation of macrophages towards M2.

## 2. Phenotypic Clustering of Dilated Cardiomyopathy Patients

Recent research from the Maastricht Cardiomyopathy Registry included 795 consecutive DCM patients who underwent extensive phenotyping, including extensive clinical data on aetiology and comorbidities, imaging, and endomyocardial biopsies [9]. On the basis of unsupervised hierarchical clustering of principal components, the authors found four mutually exclusive and clinically distinct phenogroups (PG):

PG1 included 331 patients with mild systolic dysfunction
(Mean ejection fraction: 43%  ±  9%),
PG2 included 83 patients with auto-immune disease background
PG3 included 165 patients with cardiac arrhythmias (mainly atrial fibrillation and ventricular tachycardias), including patients with genetic causes (Familial cardiomyopathy)
PG4 included 216 patients with severe systolic dysfunction (Mean ejection fraction: 23%  ±  8%)

RNA-sequencing of cardiac biopsy samples (*n* = 91) revealed a unique molecular profile per PG: pro-inflammatory (PG2, auto-immune), pro-fibrotic (PG3, arrhythmia), and metabolic (PG4, low EF) gene expression. In addition, event-free survival varied between the four phenogroups, even after adjustment for well-known clinical predictors. Using decision tree modelling, four clinical parameters (autoimmune disease, ejection fraction, atrial fibrillation, and renal function) were identified by which every DCM patient from two independent DCM cohorts could be assigned to one of the four phenogroups with corresponding outcomes.

## 3. Aetiologies of Dilated Cardiomyopathy

### 3.1. Familial Cardiomyopathy

Although a genetic basis for familial DCM is well established, the majority of cases of DCM appear to be sporadic; that is, even when the family members of patients newly diagnosed with idiopathic DCM are clinically screened, the majority of family members have no evidence of DCM; thus, patients are ultimately diagnosed with nonfamilial (sporadic) DCM. No large, multicentre study of families whose members have been systematically clinically screened for DCM and also undergone exome or genome sequencing to identify a potential genetic cause has been published to date. A total of 15–30% of patients with DCM may be diagnosed with familial DCM if their family members undertake clinical screening, according to family-based studies [3]. Historically, large multigenerational familial DCM pedigrees have served as the foundation for the majority of DCM-associated gene discovery. These multigenerational genealogies have provided statistically sound genetic evidence for the causality of variants in DCM-associated genes. The LMNA44, MYH6, MYH7, MYBPC3, TNNT2, TTN46, RBM20, SCN5A, BAG3, PSEN3 and other common DCM-causing genes were initially identified in large DCM lineage charts, along with other genes. TTN truncating mutations are a frequent cause of DCM, affecting 25% of familial cases and 18% of sporadic cases [10]. Studies suggest there is a correlation between certain DCM genotypes and left ventricular reverse remodelling (LVRR). Verdonshot and colleagues demonstrated that the TTN variant is strongly associated with higher chances of LVRR, whereas the opposite is true for the LMNA pathogenic variant [9].

### 3.2. Autoimmune Myocarditis

Autoimmune myocarditis is a condition in which cardiac muscle is damaged by self-reactive immune cells. This condition is typically associated with numerous genetic and environmental risk factors. Various systemic autoimmune diseases, such as sarcoidosis or SLE (lupus), can compromise cardiac function by a variety of mechanisms [11]. The prognosis for patients with either of these conditions is typically dismal. However, it has been demonstrated that early administration of glucocorticosteroids and immunosuppressive agents improves patients’ conditions [12]. Restoring the equilibrium between autoimmunity and immune tolerance, which is regulated by Th17 and regulatory T cells (Tregs), is one of the primary therapeutic approaches for autoimmune disease [13]. Treg cells are capable of inhibiting autoreactive T cells, and multiple studies have repeatedly identified a deficiency in the quantity or function of Treg cells in autoimmune disorders [13]. Therefore, Treg administration has been demonstrated to be a promising treatment for a variety of autoimmune disorders. Additionally, one study revealed that a modest dose of interleukin-2 can expand Tregs, thereby promoting immune tolerance [14]. Downregulating Th-17 is another promising therapeutic target for autoimmune myocarditis. Based on an experimental model of autoimmune myocarditis, Th17 was responsible for the progression of acute myocarditis with an excessive immune response [13].

### 3.3. Post-Viral Myocarditis

Myocarditis is frequently preceded by infection, with viruses being the most prevalent cause of this condition [15,16]. There are typically three phases of viral myocarditis. During the first phase, which lasts several days, the virus obtains entry and actively replicates. Therefore, the innate immune response will be triggered against these exogenous substances. During this phase, direct viral burden contributes significantly to myocardial damage. The subsequent phase revealed that an excessive immune response is the leading cause of cardiac damage. During this phase, T cell infiltration has been reported alongside an increase in fibrosis and calcification of the myocardium. In the final phase, patients may experience either remission or progression to dilated cardiomyopathy (DCM), depending on the heart’s capacity to recover from previous insults of direct viral injury and immune persistence [15,16]. By intervening in each phase of disease progression, various therapeutic targets for post-viral myocarditis can be achieved. During the viral replication phase, for instance, a potential immunotherapy for monoclonal antibodies entails shifting the immune response from Th1 to Th2 to alleviate the severity. This could be achieved by inhibiting the IP-10 signalling pathway to reduce Th1 induction and tissue recruitment [17,18].

### 3.4. Immune Checkpoint Inhibitor-Related Myocarditis

Immune checkpoint inhibitors (ICIs) are a novel type of cancer treatment that is being applied to a growing number of cancer types for example malignant melanoma, Hodgkin’s lymphoma and non small cell lung cancer to name a few. The immune modulators CTLA-4, PD-1, and PD-L1 are the targets of ICIs [19]. ICIs may, however, stimulate T cell activity against host tissues, leading to immune-related adverse events (irAEs). Myocarditis is a rare adverse reaction associated with ICIs, with incidence rates ranging from 0.1% to 2% [20]. With ICI myocarditis, there is a reduction in absolute lymphocyte count and an increase in neutrophils, both of which are associated with subsequent significant adverse cardiac events [20]. Cardiovascular magnetic resonance (CMR) utilising tissue characterisation techniques such as late gadolinium enhancement (LGE) and the presence of myocardial oedema, is the gold standard non-invasive imaging test for diagnosis and risk prediction in myocarditis of other aetiologies. LGE is present in >80% of patients with non-ICI-associated myocarditis, but in 50% of patients with ICI-associated myocarditis. The longer the interval between clinical presentation and CMR, the greater the likelihood of LGE detection [21].

CTLA-4 is a crucial regulator of T cell inhibition via multiple mechanisms, including negative signalling of B7-CD28, inhibition of IL-2 mRNA expression, and interaction with the TCR-CD3 pathway to inhibit T cell activation [22,23]. After IFN-g exposure, PD-1 and PD-L1 were highly expressed to prevent T-cell-mediated signalling on host cells. The FDA has currently approved seven ICI medications for cancer therapy. Although ICI-associated myocarditis is uncommon, its mortality rate can reach 25 to 50 percent [23]. In addition, this adverse event was reported to occur within 1 to 2 months of the initial ICI dose. The underlying pathogenesis of ICI-associated myocarditis remains unknown. As these cells shared similar antigens, one study suggested a cross-reaction of T cells towards tumour and cardiac muscle [24]. Myocarditis caused by ICI presents a spectrum of moderate to severe symptoms. The final stage of this disease, if left untreated, can be fatal, manifesting as cardiogenic shock and severe arrhythmia. Therefore, additional diagnostic procedures, such as CMR, cardiac biomarkers, and echocardiography, are advised for ICI-treated patients suspected of having myocarditis. Patients with suspected ICI-associated myocarditis should generally discontinue ICI therapy to prevent further cardiac damage. The current recommendation for ICI-induced myocarditis is the administration of glucocorticoids with continuous troponin monitoring [23]. If the patient does not respond adequately to glucocorticoids, additional treatments such as intravenous immunoglobulin, plasmapheresis, infliximab, abatacept, alemtuzumab, anti-thymocyte globulin, and mycophenolate can be considered.

## 4. Role of Fractalkine Signalling in Cardiovascular Disease

### 4.1. Fractalkine (CX_3_CL1) and Its Receptor CX_3_CR1 in Atherogenesis

**Figure 2:**
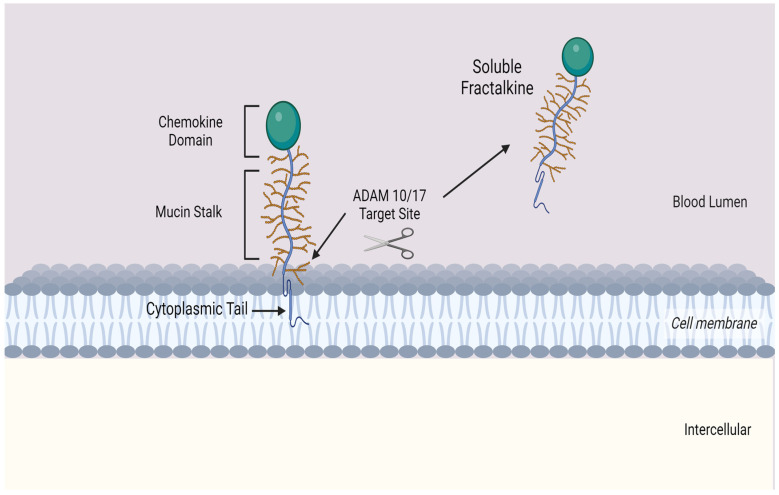
Transmembrane chemokine Fractalkine (CX_3_CL1). Graphic illustration of the transmembrane chemokine. It contains an extracellular N-domain, mucin stalk and a short cytoplasmic trail. The soluble form is generated by shedding and cleavage by metalloproteinases ADAM 10 and ADAM 17. The resulting release of the mucin-like stalk and chemokine domain acts as a chemoattractant for inflammatory cells.

Fractalkine consists of 373 amino acids and is the only member of the CX_3_C chemokine subfamily with both membrane-bound and soluble variants [25,26,27,28]. The former is an adhesion molecule with four sections: an extracellular N-terminal domain, a mucin-like stalk, a transmembrane alpha helix, and a brief cytoplasmic tail. Fractalkine can be cleaved at the juncture of its stalk and transmembrane helix my metalloproteinases ADAM 10 or ADAM 17 to produce its soluble form, which functions as a chemoattractant (see Figure 2) for monocytes, NK cells, and T cells. Fractalkine is predominantly expressed on endothelial cells, and its expression is stimulated by IFN-g and TNF-α [29]. Its G-protein-coupled receptor, CX_3_CR1, is found on inflammatory cells including NK cells, cytotoxic T cells, and monocytes [30,31], as well as on cardiomyocytes [32]. These cells are attracted along a gradient by soluble fractalkine, and endothelial cell membrane-bound fractalkine attaches to CX_3_CR1 on the surface of inflammatory cells to internalise them.

Atherogenesis begins with the sequestration of lipid-laden macrophages in the intima, a process in which fractalkine is almost certainly involved. Indeed, CX_3_CR1^−^ rodents develop fewer atherosclerotic plaques, and atherosclerotic human coronary arteries are stained with fractalkine, whereas healthy human coronaries are not [33,34]. Two polymorphisms in the CX_3_CR1 locus (V249I and T280M) have been linked to a decreased risk of atherosclerotic disease in humans [35,36,37,38,39,40,41,42,43]. Patients with the V249I polymorphism exhibited enhanced endothelium-dependent vasodilation compared to V249 homozygous patients, but comparable endothelium-independent vasodilation. It has been demonstrated that the T280M polymorphism reduces fractalkine-dependent cell–cell adhesion and induces less migration of CX_3_CR1-expressing inflammatory cells under shear stress.

Our previous research partially characterised the dynamics of CX_3_CR1 following MI and reperfusion with PCI [44]. Among CD4^+^ and CD8^+^ T cells, subdivided into CCR7^−^ and CCR7^+^ subpopulations, pre-reperfusion surface expression of CX_3_CR1 correlated strongly with the decline of that cell type at 90 min post-reperfusion, indicating that CX_3_CR1 is involved in the mechanism by which T-cells are eliminated from the blood after reperfusion. CX_3_CR1 expression is a cytotoxicity marker in both CD4^+^ and CD8^+^ T cells, and it is frequently co-expressed with perforin and granzyme B [30,45]. CMV-specific T cells (which constitute a substantial proportion of total T cells) [46,47] express a cytotoxic phenotype that includes CX_3_CR1 [48], prompting our hypothesis that CX_3_CR1-expressing T cells contribute to worse cardiovascular outcomes in CMV seropositive patients. Moreover, a study in kidney transplant recipients revealed that cytotoxic CD4^+^ T cells proliferate in response to non-specific inflammation only in CMV-seropositive patients [49,50,51], which may be explained by another group’s discovery that CMV-specific T cells express two adrenergic receptors and are therefore more sensitive to systemic inflammation [48].

### 4.2. CX_3_CR1 and Myocardial Infarction

**Figure 3:**
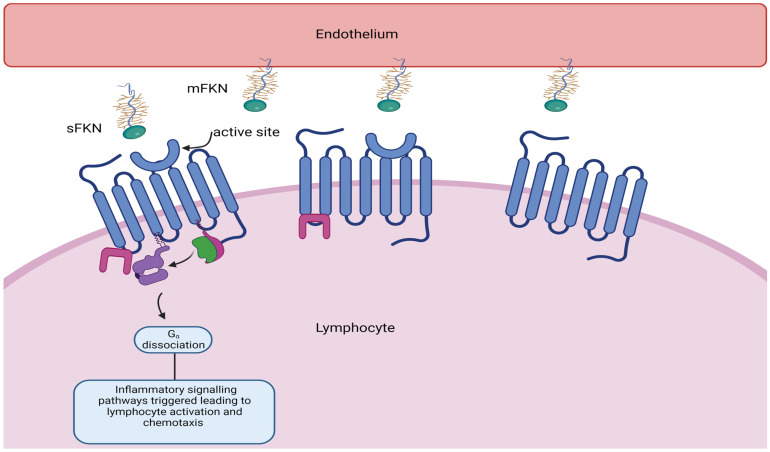
Soluble fractalkine (CX_3_CL1) binds to CX_3_CR1, its G-coupled receptor, resulting in lymphocyte activation adhesion and migration across the endothelium, resulting in tissue damage.

According to our own investigations, the CX_3_CR1 receptor is essential for the recruitment of lymphocytes that contribute to cardiac ischaemia/reperfusion injury and subsequent adverse remodelling. A subset of lymphocytes with cytotoxic properties adhere and are marginalised upon interaction with the fractalkine receptor CX_3_CR1 expressed on circulating lymphocytes (see Figure 3). It is hypothesised that lymphocyte marginalisation, myocardial damage, and myocardial inflammation can be mitigated by targeting the chemokine receptor CX_3_CR1 with inhibitors such as KAND567. Our FRACTAL phase IIa trial (ISRCTN 18402242) is presently investigating the safety and tolerability of intravenous infusion followed by oral administration of KAND567 versus placebo in 70 patients with early presenting anterior ST-elevation myocardial infarction (STEMI). To support further development in coronary heart disease and heart failure, the effectiveness of anti-inflammatory and cardioprotective effects will be examined in the same patients.

It is believed that inflammation is not only a significant factor in the development of injury, but also in the healing process and the extent of myocardial damage and function loss. Chemokines and their receptors rigorously regulate the activation and migration of immune blood cells following injury. Within 120 min of reperfusion by primary percutaneous coronary intervention (PPCI) in STEMI patients, the chemokine ligand fractalkine is activated on the endothelium and the circulating levels of subsets of T cells and monocytes expressing the fractalkine receptor (CX_3_CR1) are significantly decreased [44]. Observation of cellular retention across the myocardium (as determined by transcoronary gradients) suggests evidence for infiltration of these cells into cardiovascular tissue [44]. This immune-cell infiltration is associated with a higher risk of complications (microvascular obstruction) and a shorter three-year survival rate [44].

As it is not yet possible to track lymphocyte fate during acute myocardial infarction (MI) in patients, clinical studies have focused on the analysis of lymphocyte numbers in peripheral blood and identification of transmyocardial gradients during the acute phase of reperfusion following PPCI. A temporary decrease in lymphocytes has been identified following reperfusion [44,52]. We measured T cell gradients between the infarct-related artery and the coronary sinus, which drains venous blood from the anterior myocardium, to determine whether T cells were sequestered in the infarcted myocardium of patients [44]. A notable drop in total T cells (−3.8% ± 0.8%, *p* = 0.028) was noted in cases of anterior STEMI across this gradient when samples were collected within 45 min but not beyond this point, surmising that T cell sequestration occurs early following reperfusion.

### 4.3. Fractalkine Signalling in Heart Failure

In a study conducted by Nakayama and colleagues, higher myocardial immune activation was found to be associated with a poor prognosis for DCM [8]. Patients with higher counts of CD3^−^, CD68^−^, and CD163-positive infiltrating cells had substantially worse outcomes (*p* = 0.007, *p* = 0.011, and *p* = 0.022, respectively). The association between M2 macrophages and collagen synthesis suggests that ventricular remodelling in DCM may be associated with the phenotypic polarisation of macrophages towards M2. CX_3_CR1 and CX_3_CL1 expression is consistently upregulated in cardiac tissue from heart failure patients with various aetiologies, including end-stage DCM, according to our unpublished data. CX_3_CR1 and CX_3_CL1 gene expression has been found to be elevated in both DCM and ICM patient cardiac tissue [32]. Richter and coworkers measured the plasma levels of CX_3_CL1 in 349 patients with advanced systolic heart failure [53]. During a median of 5 years of follow-up, 56% of patients died. Fractalkine was a significant predictor of overall mortality (*p* < 0.001), with a hazard ratio of 2.8 (95% confidence interval: 1.95–3.95) for the third tertile relative to the first. This association remained significant after multivariable adjustment for demographics, clinical predictive factors, and N-terminal pro-B-type natriuretic peptide (NT-proBNP, *p* = 0.008). The predictive value of fractalkine did not differ significantly (*p* = 0.79) between patients with ischemic and non-ischemic causes of HF. Moreover, fractalkine was an independent indicator of cardiovascular mortality. Fractalkine levels in patients treated with an angiotensin-converting enzyme inhibitor were significantly reduced. The authors conclude that pro-inflammatory and immunomodulatory circulating fractalkine is an independent predictor of mortality in patients with advanced heart failure. In addition to NT-proBNP, fractalkine improves risk prediction, which may aid in the identification of high-risk patients requiring specialised care [53].

## 5. Link between CX_3_CR1 and Cytomegalovirus

**Figure 4:**
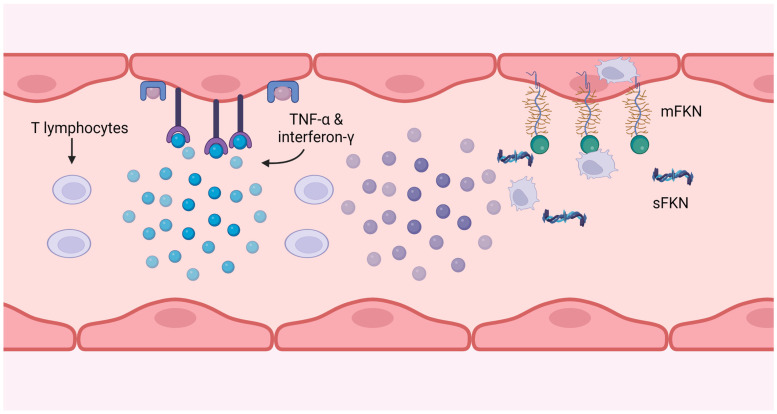
CMV-specific T cells produce interferon-g and TNF-α, which induce fractalkine expression on the endothelium. The soluble form attracts monocytes and NK cells, and the membrane-bound form catches and internalises them, removing them from the circulation.

Cytomegalovirus (CMV) is a pervasive herpes virus with a seroprevalence of greater than 60% in individuals aged 50 and older in the majority of studies [54] and 85% in those aged 80 and older [46]. CMV causes an asymptomatic or moderate initial response in immunocompetent hosts, but it is never eliminated from the body, resulting in lifelong latent infection with the possibility of reactivation [54]. Seropositivity for CMV induces significant changes in the T cell composition of the host. CMV-specific CD8^+^ and, to a lesser extent, CD4^+^ memory T cells, particularly TEMRA cells, account for a disproportionately high proportion of total T cells; this phenomenon is known as memory inflation [55,56,57]. There is evidence that memory expansion in the CD8 compartment is greater in older patients, indicating that the CMV-specific memory population continues to expand with age, but this is less evident and has been less thoroughly studied in CD4 T cells [48]. Memory inflation may be an adaptive response to suppress reactivations [58], and whether this unbalanced expansion impedes the immune system’s function remains debatable, as some contend that the CD8 compartment is sufficiently plastic to permit expansion of one phenotype without disruption of others [59]. In the presence of viral antigen, CMV-specific CD4^+^ T cells have been shown to cause endothelial injury, with more damage occurring in donors with higher frequencies of CMV-specific CD4^+^ T cells [60,61,62]. This injury is caused by the release of IFN-g and TNF-α by T cells at sufficient levels to induce endothelial cell induction of fractalkine, thereby attracting natural killer (NK) cells and monocyte-macrophages, as depicted in Figure 4. The same group demonstrated that inhibiting the fractalkine receptor, CX_3_CR1, mitigated this endothelial injury.

CMV infection transforms the T cell population towards a cytotoxic phenotype, as evidenced by the upregulation of transcription factors such as Hobit and the expression of granzymes A and B, perforin, and CX_3_CR1 [63]. Granzymes and perforin are used to lyse target cells, while fractalkine, a chemoattractant and adhesion molecule, is the CX_3_CR1 ligand. These cells were found to strongly express perforin, granzyme B, IFN-γ and TNF-α [44]. In CMV-seropositive patients, the proportion of T cells that are CD27^−^ is also considerably increased, and these cells were found to strongly express perforin, granzyme B, IFN-γ and TNF-α. This cytotoxic T cells population expands once viral load declines substantially and the infection becomes latent; it proliferates significantly more in response to CMV antigen than to other recall antigens; thus, it is CMV specific [45]. While the preponderance of CD4^+^CD27^−^ T cells in CMV-seropositive donors were CMV specific, a significantly higher proportion of CD4^+^CD27^−^ T cells were specific to other herpes virus than CMV-seronegative patients [46]. This suggests that CMV seropositivity influence extends beyond CMV-specific T cells.

CMV infection has been linked to cardiovascular disease in numerous studies, as Ji and colleagues reviewed in detail in 2012 [64]. More recently, a prospective epidemiological study in octogenarians conducted by our group revealed that CMV seropositivity and higher levels of senescent T cells such as TEMRA cells predicted worse cardiovascular outcomes [46]. Together, these factors contribute to a potential pathway in which CMV-seropositive individuals have large numbers of cytotoxic CMV-specific T cells, disproportionately TEMRA cells, and these cells respond to occasional CMV antigen presentation by inducing endothelial inflammation, thereby predisposing patients to cardiovascular disease. Additionally, relevant, animal models have demonstrated that monocytes migrate in significant numbers from the spleen to the myocardium following MI [65,66]. Monocytes are a major reservoir of CMV during latent infection [67,68], and their presence in the myocardium after an infarct could provide CMV antigen to initiate a similar pathway to that proposed above, which prolongs the inflammatory response after MI in CMV-seropositive patients, resulting in adverse remodelling.

The cardiovascular hazards associated with CMV seropositivity remain debatable. Case–control studies, such as that conducted by Siscovick [69], have failed to identify a correlation between CMV IgG and the onset of cardiovascular disease. As CMV and cardiovascular disease are so prevalent, its study design likely lacked the ability to identify this association, as acknowledged by the authors. Due to the high prevalence of CMV, a 2017 meta-analysis of prospective epidemiological studies estimated a 7–38% increased relative risk of cardiovascular disease in seropositive patients, which the authors estimated would account for 13% of the total burden of cardiovascular disease [70]. However, the effect of CMV on ventricular remodelling after STEMI has not been thoroughly studied. In a small pilot study, we demonstrated that CMV-seropositive patients exhibit worse remodelling on CMR, with ventricular dilatation 12 weeks after pPCI. This is evidence that latent CMV infection promotes a clinically significant adverse response to STEMI and pPCI, although the observed effect was small and it is imperative that this association be investigated in larger groups of patients followed for extended periods of time. A significant association after only 12 weeks may portend a significant decline in function over the course of years. Evidence from mouse models of myocardial infarction and reperfusion suggests that an abnormal inflammatory response results in detrimental remodelling. The Frangiogiannis group [71] demonstrated that CCR5 knockout mice are incapable of recruiting regulatory T cells to the infarcted myocardium and exhibit poorer remodelling, whereas Cochain and colleagues demonstrated that the decoy receptor D6, which internalises and deactivates pro-inflammatory chemokines, is required for healthy remodelling in the infarcted murine heart [72]. FoxP3^−^CD4^+^ T cells substantially contribute to age-related myocardial inflammation in mice, as demonstrated by our collaborators [73]. Further research revealed that CMV-seropositive patients exhibit evidence of accelerated immune ageing following myocardial infarction, which appear to be related to impaired myocardial healing [74,75].

## 6. Conclusions

The evidence base and existing literature suggest that upregulation of fractalkine/CX_3_CR1, together with CMV seropositivity and consequent migration of immune and inflammatory cells, is associated with worse outcomes in patients with inflammatory conditions including dilated cardiomyopathy. We hypothesise that dilated cardiomyopathy, whether acquired secondary to infection, autoimmunity, drugs and toxins or familial in origin, may lead to cardiac inflammation and fibrosis, resulting in adverse remodelling through the interplay of these factors. Robust observational studies are required to identify a subgroup of at-risk patients with dilated cardiomyopathy through in-depth phenomapping, genotyping, evaluation of immune status, and viral presence, which in turn may inform future drug trials utilising immune modulators to target this pathway.

## Figures and Tables

**Table 1:** Causes of Dilated Cardiomyopathy.

Genetic Causes	LMNA44, MYH6, MYH7, MYBPC3, TNNT2, TTN46, RBM20, SCN5A, BAG3, PSEN3	
Infections	Viruses	*Adenovirus* spp., *Coronavirus* spp., *Coxsackievirus* spp. (groups A and B), *Cytomegalovirus* spp., Dengue virus, *Echovirus* spp., Epstein-Barr virus, Hepatitis B virus, Hepatitis C virus, Herpes Simplex Virus, Human Herpesvirus 6, HIV, Influenza A and Influenza B viruses, Mumps rubulavirus, parvovirus (B19), poliovirus, Rabies virus, Respiratory syncytial virus, Rubella virus, Measles virus, and Varicella- zoster virus
	Bacteria	ß-haemolytic streptococci, *Borrelia burgdorferi*, *Brucella* spp., *Campylobacter jejuni*, *Chlamydia* spp., *Clostridium* spp., *Corynebacterium diphtheriae*, *Neisseria* spp., *Haemophilus influenza*, *Legionella pneumophila*, *Listeria monocytogenes*, *Mycoplasma pneumonia*, *Neisseria meningitidis*, Salmonella (Berta and Typhi), *Streptococcus pneumonia*, *Staphylococcus* spp., and *Treponema pallidum*
	Protozoa	*Entamoeba histolytica*, *Leishmania* spp., *Plasmodium vivax*, *Plasmodium falciparum*, *Toxoplasma gondii*, and *Trypanosoma cruzi*
	Helminths	*Taenia* spp., *Echinococcus* spp., *Schistosoma* spp., *Toxocara* spp., and *Trichinella* spp.
	Fungi	*Actinomyces* spp., *Aspergilus* spp., *Coccidioides immitis*, and *Cryptococcus neoformans*
Autoimmunity	Systemic sclerosis, rheumatoid arthritis, systemic lupus erythematosus, dermatomyositis, sarcoidosis, Dressler syndrome, post-cardiotomy syndrome, post-infectious autoimmune disease, and post-radiation autoimmune disease	
Toxin exposure	Alcohol, amphetamines, anthracyclines, cannabis, catecholamines, cocaine, 5-fluorouracil, lithium, heavy metals (cobalt, lead, and mercury), and carbon monoxide	
Metabolic or Endocrine	Cushing disease, hypothyroidism, hyperthyroidism, phaeochromocytoma, chronic hypocalcaemia, hypophosphataemia, and inborn errors of metabolism such as mitochondrial diseases and nutritional deficiency (carnitine, thiamine, and selenium)	
Pregnancy	Peripartum cardiomyopathy	

## Data Availability

Data sharing not applicable.
